# The influence of trophic status and seasonal environmental variability on morpho-functional traits in tropical man-made shallow lakes

**DOI:** 10.1007/s10661-022-10091-y

**Published:** 2022-06-16

**Authors:** Rayane F. Vanderley, Vanessa Becker, Renata Panosso, Kemal A. Ger, Judit Padisák

**Affiliations:** 1 Research Group of Limnology, Centre of Natural Sciences, University of Pannonia, Egyetem u. 10, Veszprém, 8200 Hungary; 2 Laboratory of Water Resources and Sanitation, Federal University of Rio Grande Do Norte (UFRN), Natal, RN 59072-970 Brazil; 3 Department of Microbiology and Parasitology, Federal University of Rio Grande Do Norte (UFRN), Natal, RN 59072-970 Brazil; 4 Department of Ecology, Federal University of Rio Grande Do Norte (UFRN), Natal, RN 59072-970 Brazil

**Keywords:** Morphological traits, Functional approach, Dryness, Eutrophication, Semiarid, Harmful algal blooms

## Abstract

In the tropics, seasons are delimitated by the extent of rainfall resulting in seasonal differences in water parameters shaping phytoplankton community dynamics. Dry periods can intensify eutrophication and often result in seasonal or even perennial cyanobacterial dominance. This study was developed to evaluate phytoplankton response to trophic state and seasonal differences of environmental filters (dry and rainy periods) using the morphology-based functional groups (MBFG) approach. We also aimed at identifying environmental thresholds of each MBFG dominance in six man-made lakes located in the tropical semiarid region of Brazil. Our results showed clear MBFG association with lakes’ trophic states. The dominant groups in mesotrophic conditions were members of MBFGs V (unicellular flagellates) and VI (non-flagellated with a siliceous exoskeleton), and in meso-eutrophic MBFG IV (medium size without specialized traits) dominated. Conversely, MBFG VII (with mucilage and aerotopes) and VIII (nitrogen-fixing cyanobacteria) dominated mostly under eutrophic conditions, though linked to shallower euphotic zones. Light and phosphorous were the most important environmental thresholds associated with MBFG’s dominance. Overall, most of the lakes displayed seasonal differences in environmental filters. In contrast to what was expected, the rainy season was associated with higher nutrients, suspended solids, and reduced euphotic depth compared to the dry season. Our results, overall, show that the effects of seasonality varied across lakes and highlight eutrophication as the main environmental factor for MBFG selection suggesting reduced seasonality effects during dry years in the tropics.

## Introduction

Phytoplankton is an extremely diverse polyphyletic group of biota comprising organisms with a variety of functional traits (Litchman & Klausmeier, 2008). Species sharing trait properties are influenced similarly by environmental filters and may similarly affect ecosystem functioning, even if they are not related taxonomically (Kruk et al., 2020; Salmaso & Padisák, 2007). Based on species’ adaptive strategies (i.e., conferred by the functional traits), it is possible to identify and understand the environmental drivers of phytoplankton assemblage dynamics (Brasil & Huszar, 2011; Reynolds et al., 2002; Salmaso & Padisák, 2007; Salmaso et al., 2015). Thus, when the habitat template and environmental thresholds of functional groups’ dominance are defined, the link between functional traits and environmental variables allows for the assessment of lake conditions based on the pool of species (Kruk & Segura, 2012; Kruk et al., 2017, 2020; Reynolds et al., 2002).

Artificial eutrophication is the most common cause of freshwater quality impairment worldwide (Le Moal et al., 2019). The subsequent phytoplankton overgrowth, commonly by bloom-forming cyanobacteria, has major consequences on biodiversity, ecosystem functioning, and human health (Carpenter & Cottingham, 1997; Cottingham et al., 2015; Ibelings et al., 2007). Besides eutrophication, other environmental pressures influence phytoplankton assemblage structure. For example, warming and extreme weather events, including storms and drought, may also favor the global expansion of cyanobacterial blooms (Giani et al., 2020; Havens et al., 2019; Kasprzak et al., 2017; Moss, 2011; Paerl & Huisman, 2008; Paerl et al., 2020; Stockwell et al., 2020).

In tropical regions, the annual cycle of precipitation (i.e., rainy and dry seasons) promotes changes in chemical and physical water parameters leading to seasonal differences in phytoplankton assemblage composition (Azevedo et al., 2020; Bortolini & Bueno, 2017; Braga & Becker, 2020; Rangel et al., 2016). Dry periods reduce the water level and have been generally associated with increased eutrophication, water residence time, phytoplankton biomass, mainly cyanobacteria, and decreased water transparency (Aldridge, 2014; Braga et al., 2015; Brasil et al., 2016; Havens et al., 2016, 2019; Medeiros et al., 2015; Mosley, 2015; Naselli-Flores, 2003; Padisák et al., 1999; Rocha Junior et al., 2018). Episodic rains may increase nutrient availability via short-term runoff events, although dilution effects are expected during long rainy periods (Brasil et al., 2016; Jargal et al., 2021). Thus, the increase in water level and subsequent decrease in nutrient concentrations promotes a reduction in phytoplankton biomass, leading to higher water transparency (Braga & Becker, 2020; Brasil et al., 2016; Stockwell et al., 2020). Moreover, the effects of rainy and dry periods on lakes depend on characteristics such as their duration and intensity, along with lakes’ characteristics, such as depth, volume, and trophic status (Costa et al., 2016; Medeiros et al., 2015; Stockwell et al., 2020).

Trophic status and seasonality (i.e., dry and rainy periods) play an important role in resource availability and shaping the composition of phytoplankton dynamics (Bortolini & Bueno, 2017; Naselli-Flores, 2000; Pacheco et al., 2010). Therefore, functional approaches have been widely used and recommended to understand phytoplankton responses to environmental variability, as they are often more reliable than the taxonomic approach (Kruk et al., 2010; Padisák et al., 2009; Reynolds et al., 2002; Salmaso & Padisák, 2007). Hence, since the first attempts to sort phytoplankton species into functional groups (Margalef, 1978; Padisák & Reynolds, 1998; Reynolds, 1984), multiple efforts have been refined towards improving and validating such models (Derot et al., 2020; Kruk et al., 2010; Padisák et al., 2009; Reynolds et al., 2002; Salmaso & Padisák, 2007). The selection of the specific functional approach depends on the ecological question and may include morphological, physiological, and ecological features such as phenology, and taxonomy when necessary (Salmaso et al., 2015). Morphological traits are often used as they can easily be measured and have a clear relation with phytoplankton ecological function (i.e., reproduction, resource acquisition, and predator avoidance); thus, it is possible to implicate their function in the environment (Kruk & Segura, 2012; Kruk et al., 2010; Litchman & Klausmeier, 2008; Litchman et al., 2010; Naselli-Flores, 2014; Naselli-Flores et al., 2007; Segura et al., 2013).

A functional approach, proposed by Kruk et al. (2010), is exclusively based on easily observable morphological characteristics, such as cell size and volume, presence of mucilage, flagella, aerotopes, heterocytes, and siliceous structure. These morphological traits were correlated to functional properties and abundance, resulting in the classification of seven groups and subsequently their habitat templates (Kruk & Segura, 2012; Kruk et al., 2010). Later, the classification was updated to eight groups by defining nitrogen-fixing cyanobacteria species as a separate group (Reynolds et al., 2014). Morphologically based functional groups (MBFGs) have a close relationship to environmental conditions, and although they have been efficiently applied to a variety of freshwater environments, in some tropical regions, relatively few studies have been reported, such as in Africa and Asia (Gebrehiwot et al., 2017; Hu et al., 2013; Lu et al., 2021). Additionally, the MBFG approach has the advantage of being more easily applicable over a large number of samples and is suitable for environments with different stable states (Izaguirre et al., 2012).

In the present study, we applied the MBFG approach to link environmental filters of tropical shallow lakes with phytoplankton community structure, using data from six man-made lakes in the Brazilian semiarid region as a model. Only a few cases have applied the MBFG approach in tropical semiarid regions with prolonged droughts. This region is sensitive to extremes of climate variability, and climate change projections predict a further increase in rainfall deficit and aridity for the next century (Marengo & Bernasconi, 2015; Marengo et al., 2020). Although the annually recurrent dry period is part of the hydrological cycle in this region, its intensity has been increasing along with the incidence, magnitude, and persistence of toxic or potentially toxic cyanobacterial blooms (Bouvy et al., 2000, 2003; Marengo et al., 2017; Moura et al., 2018; Pedrosa et al., 2020). Furthermore, the consequences of intensified dryness in such regions on the dynamics and structure of phytoplankton have remained largely unknown. Drought could favor either cyanobacterial dominance (Bouvy et al., 2003; Brasil et al., 2016; McGregor & Fabbro, 2000) or mixotrophic organisms and diatoms depending on drought intensity and lake depth (Costa et al., 2016, 2019; Crossetti et al., 2019; Medeiros et al., 2015). Overall, comprehending the associated effects of drought and eutrophication is a major challenge for understanding phytoplankton dynamics and managing cyanobacterial blooms under the global change scenario (Paerl et al., 2020).

This work is based on our previous research characterized by reduced water depth and eutrophication, where potential environmental filters regulated the switch between the two dominant phytoplankton species, *Raphidiopsis raciborskii* (Woloszynska) Aguilera, Berrendero Gómez, Kastovsky, Echenique, and Salerno and *Microcystis aeruginosa* (Kützing) Kützing during a perennial cyanobacteria dominance (Vanderley et al., 2021)*.* In the present study, we extend the focus to assess the environmental filters influencing the phytoplankton assemblage using the morphology-based functional groups (MBFG) approach. Specifically, our aims were to (1) evaluate MBFG’s response to trophic state, (2) determine the environmental filters threshold linked with each MBFG’s dominance, and (3) assess the influence of dry and rainy seasons on environmental filters and MBFG’s selection in six semiarid shallow man-made lakes. Because lakes with similar trophic states are expected to display similar morpho-functional trait selection regarding phytoplankton, we hypothesized that (1) morpho-functional traits known to overcome the light limitation, i.e., high surface/volume (s/v) and maximum linear dimension (MLD) and buoyant properties (aerotopos and mucilage), will be favored in eutrophic environments, and traits known to overcome nutrient limitation, i.e., flagella, mixotrophy, and heterocytes, will be favored in mesotrophic lakes, and (2) dry and rainy seasons will have a contrasting relationship on water depth, the concentration of nutrients and solids, and light availability selecting distinct MBFGs. Finally, we discuss the implications for the use of MBFG as a tool for biomonitoring purposes in lakes aimed for multiple uses where persistent cyanobacteria blooms occur.

## Material and methods

### Study area and climatological scenario

**Fig. 1:**
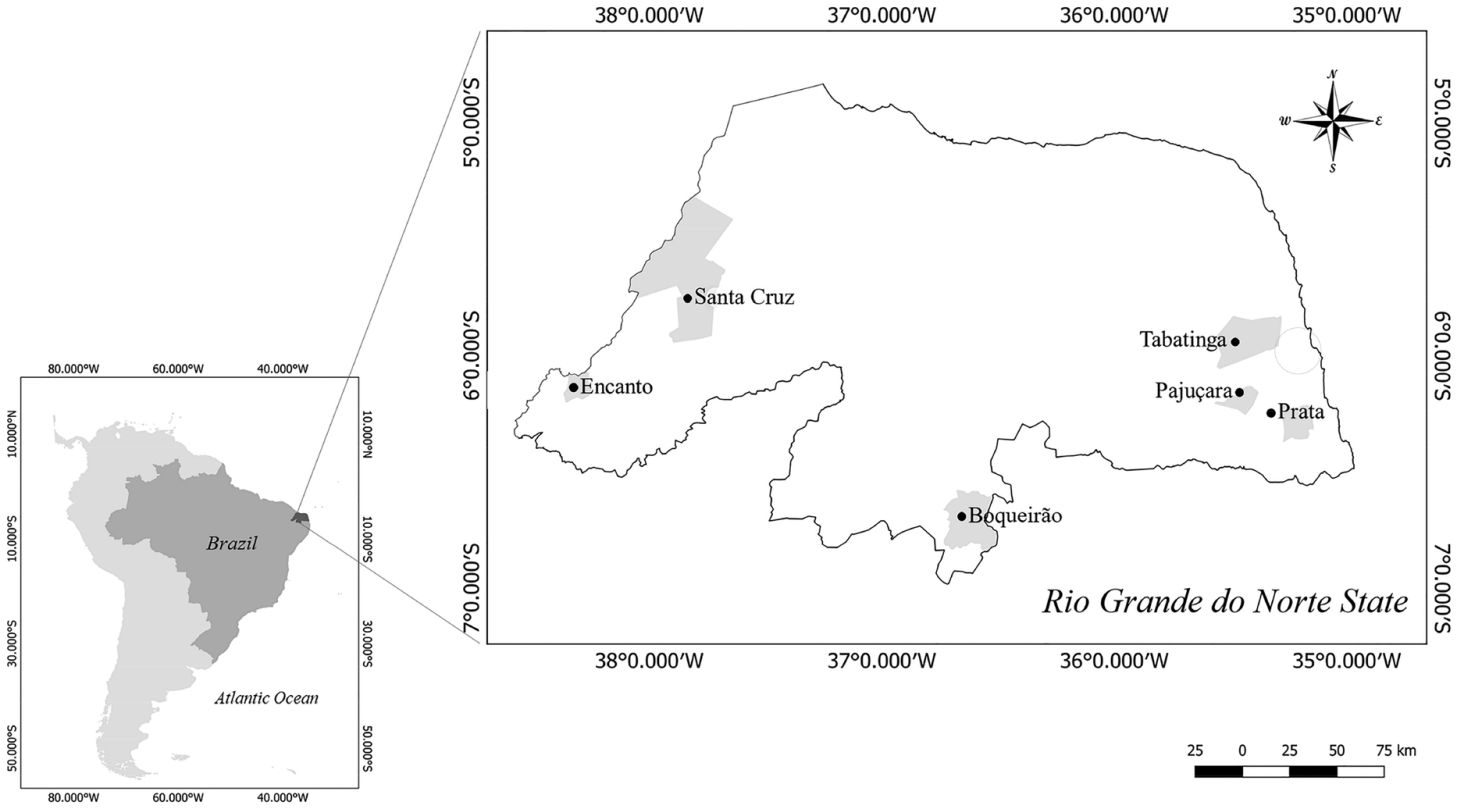
Map outline of South America showing Rio Grande do Norte state within Brazilian territory, and the location of the six studied lakes

Data from the phytoplankton and environmental filters were collected from six man-made lakes located in a semiarid region in Rio Grande do Norte state, in the northeast region of Brazil (Fig. 1). The six lakes differed in storage level (%), maximum, and current depth and covered a trophic state gradient from mesotrophic to eutrophic (Table 1). The trophic states of the lakes were previously determined based on total phosphorus and chlorophyll-a thresholds as proposed for semiarid lakes (). All lakes were vertically well-mixed during the study and the eutrophic ones, Encanto, Boqueirão, and Pajuçara exhibited perennial dominance of cyanobacteria (further details in Vanderley et al., 2021). These lakes were created to retain water during the rainy season to overcome water shortages in the region during the dry months. The lakes have been providing several other services, such as water for crop irrigation, subsistence, recreational fishing, and livestock maintenance and, for Santa Cruz, and Boqueirão, domestic water supply.

**Table 1:** Morphometric variables of the six lakes included in the study. Storage level (%) means the percent of the maximum volume retained in the lake in 2017. Maximum depth refers to the depth of the lakes in full capacity and current depth is the average of all measures done in 2017. Data on storage level and maximum depth were not available (NA) for Pajuçara Lake. Trophic state based on Thornton & Rast (1993). Data source: State Department of the Environment and Water Resources (SEMARH) and Vanderley et al. (2021)

	**Storage level (%)**	**Maximum depth (m)**	**Current depth (m)**	**Trophic state**
Santa Cruz	24.7	57.5	16.6	Mesotrophic
Prata	81.9	19.5	3.9	Mesotrophic
Tabatinga	11.8	27.8	5	Meso-Eutrophic
Encanto	74.0	16.9	5.9	Eutrophic
Boqueirão	33.2	29.0	5	Eutrophic
Pajuçara	NA	NA	4.5	Eutrophic

Lakes are located in a warm semiarid region (with mean annual air temperatures ~ 26.5 °C, annual rainfall is below 650 mm, aridity index (precipitation/potential evapotranspiration) below 0.50, and a drought risk index (days of soil moisture deficit from water balance) of above 60% (Superintendência do Desenvolvimento do Nordeste, Sudene, 2017; Marengo et al., 2020). In this region, the seasonal cycle is distinguished by two major annual periods: the rainy season, with rainfall, concentrated typically from January to June, and the dry season with a negative water balance during the rest of the year. Although drought is a natural phenomenon in this region, it is becoming drier, and the mean annual precipitation has already decreased by half from the 1980s to the 2000s (Marengo et al., 2017). This region faced a prolonged drought during the period 2012–2016 with rainfall deficit varying between 20 and 60% below the historical mean (Marengo et al., 2017, 2018), which refers to the previous years to our sampling period in 2017.

### Environmental filters sampling and analysis

**Fig. 2:**
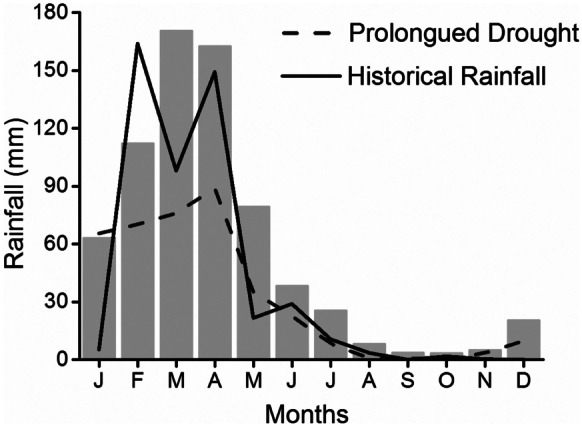
Monthly rainfall accumulated between January and Dec 2017 (gray bars); historical monthly average rainfall between 1963 and 2011 (solid black line); and monthly average rainfall during the prolonged drought from 2012 to 2016 (dashed black line) in the Rio Grande do Norte semiarid region. (Data source: Agricultural Research Company of Rio Grande do Norte State—EMPARN)

In order to describe the rainfall scenario in the studied lake area, we analyzed monthly rainfall data during the sampling period (January to December 2017), the historical time series prior to the sampling period, comprising the years 1963 to 2011, and during the prolonged drought, from 2012 to 2016 (Cunha et al., 2019). Pluviometric data from two meteorological stations located in the vicinity of the lakes were provided by the Agricultural Research Company of Rio Grande do Norte (EMPARN). The averages of these data were used to characterize the rainfall in the region (Fig. 2).

This study was conducted every month between January and December 2017, except in June in Santa Cruz and in April in Boqueirão due to technical problems resulting in a total of 70 samples. Integrated water samples from the upper 2 m of the water column were taken with a PVC tube at a short distance from the dam, which corresponds to the deepest site of each lake. Depths were measured at each site with a graduated anchored rope; this procedure was repeated three times, and the average value was used to obtain maximum depth, *Z*_max_ (m). Light availability in the water column was estimated by a Secchi disk extinction depth. The euphotic zone depth, *Z*_eu_ (m) was determined as 2.7 times the Secchi depth measurements (Cole, 1994). Light availability in the water column was estimated by using the ratio *Z*_eu_:*Z*_max_ as a proxy (Jensen et al., 1994). Water temperature was measured at the surface and the bottom of the water column.

Chlorophyll-a (Chla; µg L^−1^), total suspended solids (TSS; mg L^−1^), volatile suspended solids (VSS; mg L^−1^), and fixed suspended solids (FSS; mg L^−1^) were analyzed in the particulate fraction of water samples retained on GF/C (1.2 μm) filters. Solids were quantified through gravimetry (Chanlett, 1947) and Chla was detected by means of 95% ethanol extraction followed by spectrophotometric measurements (Wintermans & De Mots, 1965; Jespersen & Christoffersen, 1987; see Vanderley et al., 2021 for details). Total nitrogen (TN; µg L^−1^), total dissolved nitrogen (TDN; µg L^−1^), total carbon (TC; µg L^−1^), and total dissolved carbon (TDC; µg L^−1^) concentrations were determined using standard techniques in a SHIMADZU TOCVCPN sampler with the SSM-5000A solid sample combustion unit, while total phosphorus (TP; µg L^−1^) and soluble reactive phosphorus (SRP; µg L^−1^) were quantified colorimetrically (Vanderley et al., 2021).

### Phytoplankton sampling and analysis

Phytoplankton samples were fixed with acetic Lugol’s solution. Samples for chemical and phytoplankton analysis were collected, stored, and preserved as described in Vanderley et al. (2021). Lugol-fixed integrated samples were settled at appropriate volumes in a sedimentation chamber and phytoplankton units (cells, filaments, or colonies) were enumerated in random fields with an inverted microscope according to the Utermöhl method (Uhelinger, 1964; Utermöhl, 1958). Enumerations (individuals mL^−1^) were performed until counts reached at least 100 individuals of the most frequent species (*p* < 0.05; Lund et al., 1958). Phytoplankton was identified to species level whenever possible; an optical light microscope was also used when necessary (× 400 magnification).

Organism dimensions of 40–60 phytoplankton specimens were measured for each sample, and population biovolume (mm^3^ L^−1^) was estimated from the specific individual volume, assessed from geometric models (Fonseca et al., 2014; Hillebrand, 1999), multiplied by the abundance of individuals. Then, specific biomass (mg L^−1^) was converted considering the fresh weight is equivalent to a mass of 1 mm^3^ L^−1^ = 1 mg L^−1^ (Wetzel & Likens, 2000). Species representing more than 5% of the total biomass per sample were affiliated into eight morphologically based functional groups (MBFG). Seven of them were proposed by (Kruk et al., 2010): small organisms with high s/v (MBFG I); small flagellated organisms with siliceous exoskeleton (MBFG II); large filaments with aerotopes (except nitrogen-fixing species) (MBFG III); organisms of medium size without specialized traits (MBFG IV); unicellular flagellated organisms (MBFG V); non-flagellated organisms with siliceous exoskeleton (MBFG VI); and presence of mucilage along with aerotopes (MBFG VII). Moreover, we included a group of nitrogen-fixing cyanobacteria (MBFG VIII), as proposed by Reynolds et al. (2014).

### Data analysis

Principal component analysis (PCA) was applied to evaluate the patterns of the limnological variables (*Z*_max_, *Z*_eu_, TP, TN, TC, and Chla) among lakes and seasons (dry and rainy). For the ordination analysis, the data were scaled. The variables included in the PCA were previously indicated as the most important for the phytoplankton (Vanderley et al., 2021). Considering the parametric assumptions were not met, the Mann–Whitney *U* test was applied to test the differences among dry and rainy seasons regarding abiotic and biotic data, while Kruskal–Wallis test was performed to evaluate significant variances of each MBFG among trophic states. All analyses above were conducted using the function of the R package *vegan* (Oksanen et al., 2020; Team, 2021). Following, we did a classification tree (CART) (De’ath & Fabricius, 2000) using the package *rpart* in R (Therneau & Atkinson, 2019; Team, 2021) to identify the environmental thresholds leading to the dominance of each MBFG according to Kruk & Segura (2012). In order to evidence dominance by a group on the lake, we included only MBFG with relative biomass equal to or above 70% of the total phytoplankton biomass (Naselli-Flores et al., 2003), this way, each sample had only one MBFG as an outcome. All environmental filters measured were included in the classification tree, except the dissolved nutrient forms due to their high variability and their dependence on phytoplankton and bacteria consumption, which would have confused to establish cause-effect relationships (Kruk & Segura, 2012).

## Results

### Environmental and climate characteristics

**Fig. 3:**
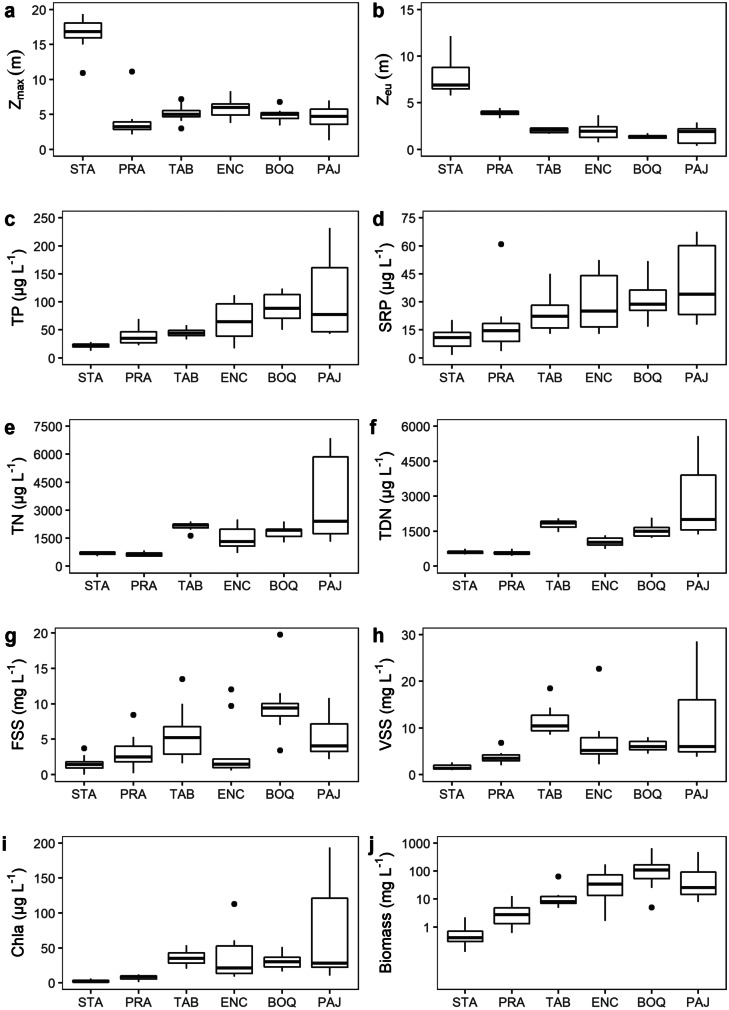
Boxplot of limnological variables of each lake sampled monthly between January and Dec 2017; mesotrophic; STA, Santa Cruz; PRA, Prata; meso-eutrophic; TAB, Tabatinga; eutrophic; ENC, Encanto; BOQ, Boqueirão; PAJ, Pajuçara. **a** maximum depth, **b** euphotic zone, **c** total phosphorous, **d** soluble reactive phosphorous, **e** total nitrogen, **f** total dissolved nitrogen, **g** fixed suspended solids, **h** volatile suspended solids, **i** chlorophyll-a, and** j** phytoplankton biomass (*y*-axis in log scale). Bands inside the boxplots represent their medians and vertical bars represent minimum and maximum values. Black dots indicate values that fall outside 1.5 times the interquartile range. Data from Vanderley et al. (2021)

In 2017, the annual rainfall was 31% lower than the historical average (1963–2011) (31%) but it was 21% above the prolonged drought average (2012–2016) (Fig. 2). With only a single exception, the water temperature was always equal to or higher than 25 °C, but never above 31 °C, and an average ranged from 27 to 28 °C (data not shown). The average maximum depth ranged from 3.9 m at Prata Lake to 16.6 m at Santa Cruz, while the other lakes had maximum depths of approximately 5.0 m (Fig. 3a).

As the lakes displayed a gradient of trophic status, we present them in this section accordingly: from the mesotrophic lakes, Santa Cruz and Prata, to the most eutrophic one, Pajuçara. Accordingly, there was an increase in TP, SRP, and phytoplankton biomass towards the most eutrophic, Pajuçara Lake (Fig. 3c, d, j). An inverse pattern was observed for light availability in the water column; the Z_eu_ was deeper in the mesotrophic lakes, Santa Cruz, while the eutrophic lakes were characterized by mean values equal to or below 2.2 m (Fig. 3b). Prata was the only lake where the entire water column was generally clear during most of the study, with *Z*_eu_:*Z*_max_ ≥ 1 in eight months out of a total of twelve months. For all other lakes in all sampled months *Z*_eu_:*Z*_max_ < 1 (data not shown).

The mesotrophic lakes (Santa Cruz and Prata) also showed lower concentrations of TN, TDN, FSS, VSS, and Chla (Fig. 3e, f, g, h, i). For the eutrophic lakes, the highest concentrations of TN and TDN were recorded at Pajuçara Lake (Fig. 3e, f), while Pajuçara presented the highest for Chla (Fig. 3i). SRP was perennially above 3 µg L^−1^, except for one month in Santa Cruz, when SRP was 1.66 µg L^−1^ (Fig. 3d) and TDN was always above 450 µg L^−1^ (Fig. 3f).

**Fig. 4:**
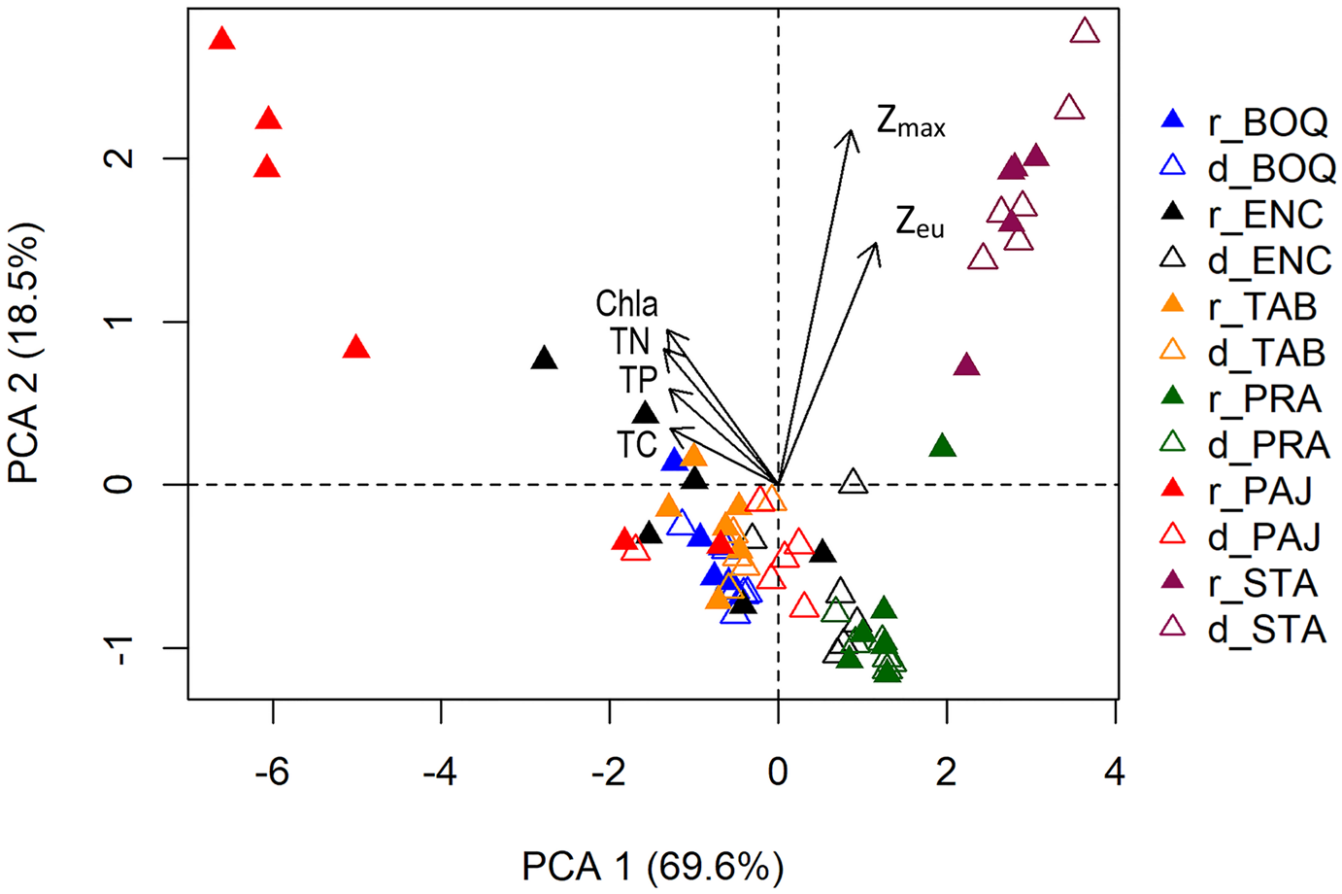
Principal component analysis (PCA) of the limnological variables studied in the six man-made lakes studies during the rainy (full triangle) and dry (empty triangle) seasons. Lakes: STA, Santa Cruz; PRA, Prata; TAB, Tabatinga; ENC, Encanto; BOQ, Boqueirão; PAJ, Pajuçara. Variables: *Z*_eu_, euphotic zone depth; *Z*_max_, maximum depth; TP, total phosphorus; TC, total carbon; TN, total nitrogen; Chla, chlorophyll-a

The PCA showed the ordination of sampling units regarding lakes and seasonality (rainy and dry periods) (Fig. 4). The two first axis explained 88.1% of the variability (axis 1 = 69.6% and axis 2 = 18.5%). For axis 1, the most important variables were TN (− 0.45), Chla (− 0.44), TP (− 0.43), and TC (− 0.42). While for axis 2, *Z*_max_ (0.72) and *Z*_eu_ (0.49) were the most important variables. The first axis presented mostly a trophic tendency; on the positive side were the samples from dry and rainy periods of the mesotrophic lakes (Santa Cruz and Prata) and samples from the dry period of Encanto and Pajuçara with low concentrations of TN, Chla, TP, and TC. On the other hand, on the negative side were the samples from the dry and rainy periods in Tabatinga and Boqueirão (meso-eutrophic and eutrophic) and samples from the rainy period of Encanto and Pajuçara with a higher concentration of these variables. Axis 2 indicated samples from Santa Cruz with the highest *Z*_eu_ and *Z*_max_ compared with the other sample units.

### Phytoplankton community structure

**Fig. 5:**
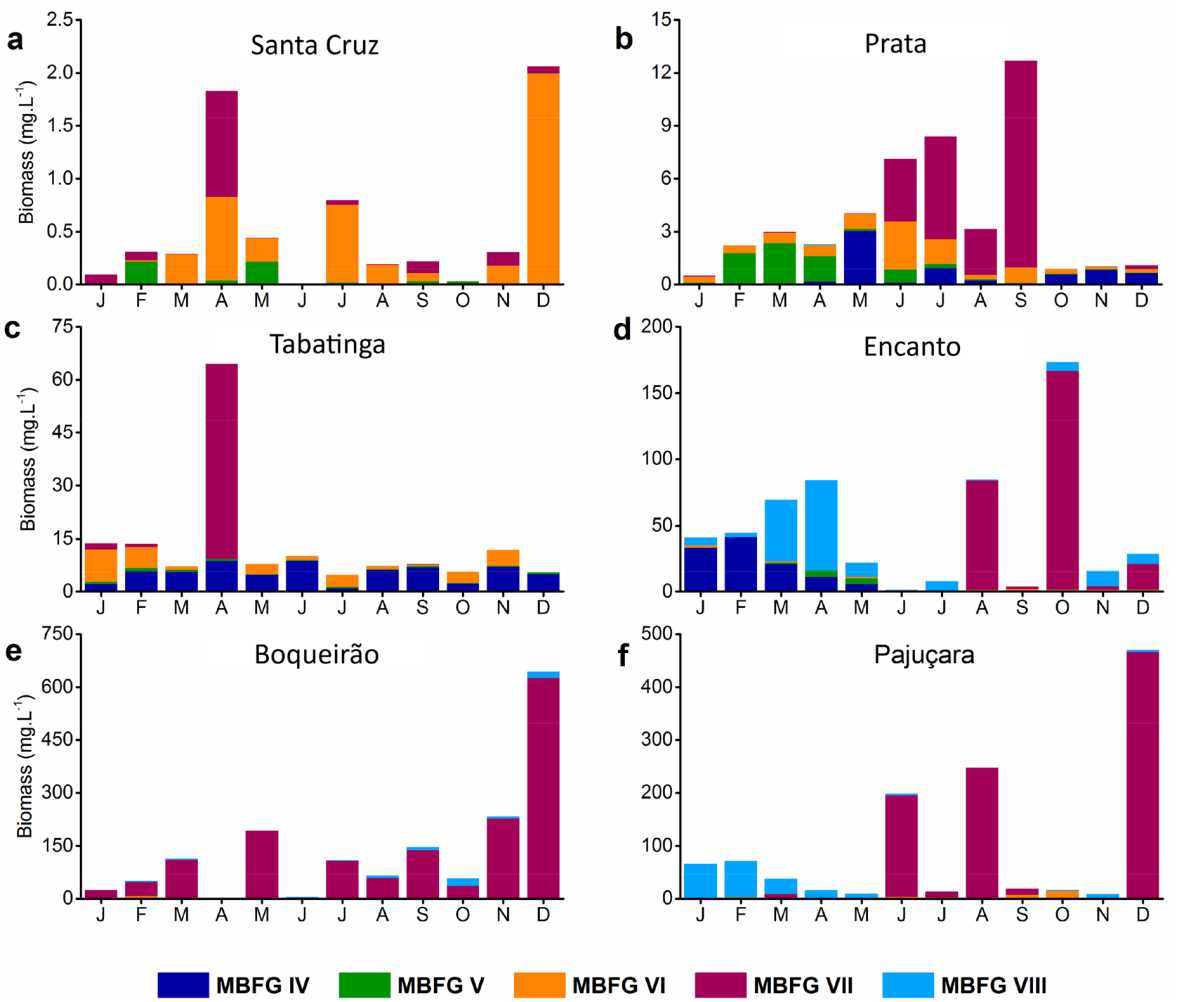
**a**–**f** Total phytoplankton and MBFG biomass composition for each studied lake during the year 2017 (January to December). Data were not available for Santa Cruz in June and Boqueirão in April. Note the different *y*-axis scales for each lake

A total of 343 taxa were recorded during the study, of which 25 taxa for the descriptor species were grouped into five MBFGs (IV, V, VI, VII, VIII; Table 2). Of the 70 samples, 50 presented monodominance by a single MBFG (≥ 70% of total biomass). The mesotrophic Santa Cruz Lake had the lowest phytoplankton biomass recorded among the lakes and was dominated by MBFG VI most of the time, yet MBFGs V and VII also contributed to the community (Fig. 5a). The dominant group at the mesotrophic Prata Lake alternated through time and was mainly composed of MBFGs IV and VII, yet MBFGs V and VII also contributed to the community (Fig. 5b). Tabatinga Lake (meso-eutrophic) was mostly co-dominated by MBFG IV and VI, except in April, when MBFG VII achieved the highest biomass observed for this lake (Fig. 5c). At the eutrophic Encanto Lake, MBFGs VI, VII, and VIII were the most representative phytoplankton functional groups (Fig. 5d; Table 2). Phytoplankton at Boqueirão Lake (eutrophic) was dominated by MBFG VII nearly all year (Fig. 5e). Pajuçara Lake (eutrophic) was dominated mainly by MBFG VII and VIII (Fig. 5f). Moreover, MBFG VII contributed most to the total biovolume, representing the highest biomass achieved in all lakes, except for Santa Cruz lake, where MBFG VI achieved the highest biomass (Fig. 5).

**Table 2:** Representative species of each lake and their respective morpho-functional groups (MBFG)

**MBFG**	**Morphology**	**Species**
**IV**	Organisms of medium size lacking specialized traits	*Coelastrum reticulatum* (Dangeard) Senn
		*Monoraphidium minutum* (Nägeli) Komárková-Legnerová
		*Planktolyngbya limnetica* (Lemmermann) Komárková-Legnerová and Cronberg
		*Pseudanabaena catenata* Lauterborn
		*Pseudanabaena* sp.
**V**	Unicellular flagellates of medium to large size	*Trachelomonas armata* Ehrenberg
		*Trachelomonas* sp.
		*photosynthetic flagellates*
		*Phacus* sp*.*
		*Peridinium* sp*.*
		*Cryptomonas* sp.
**VI**	Non-flagellated organisms with siliceous exoskeletons	*Cyclotella* sp*.*
		*Aulacoseira granulata* (Ehrenberg) Simonsen
		*Aulacoseira granulata* var. *angustissima* (O. Müller) Simonsen
**VII**	Large mucilaginous colonies	*Aphanocapsa* cf*. nubilum* Komárek and H.J.Kling
		*Aphanocapsa delicatissima* West and G.S.West
		*Aphanocapsa incerta* (Lemmermann) G. Cronberg and Komárek
		*Aphanocapsa* sp.
		*Botryococcus braunii* Kützing
		*Microcystis panniformis* Komárek
		*Microcystis aeruginosa* (Kützing) Kützing
		*Oocystis* sp.
		*Chroococcus* sp.
**VIII**	Nitrogen-fixing cyanobacteria	*Raphidiopsis raciborskii* (Woloszynska) Aguilera, Berrendero Gómez, Kastovsky, Echenique and Salerno

### MBFG response to trophic status and environmental thresholds

Regarding MBFG variability, all MBFG significantly differed according to the lake’s trophic state (IV, VII, and VIII *p* < 0.000; V *p* < 0.005; VI *p* < 0.05). MBFG V was associated exclusively with mesotrophic conditions, on the other hand, MBFG VIII was exclusively from eutrophic conditions. MBFG VI dominated in mesotrophic and meso-eutrophic conditions. MBFG IV dominated mostly in meso-eutrophic conditions. MBFG VII was the only one present in all lakes regardless of trophic state.

**Fig. 6:**
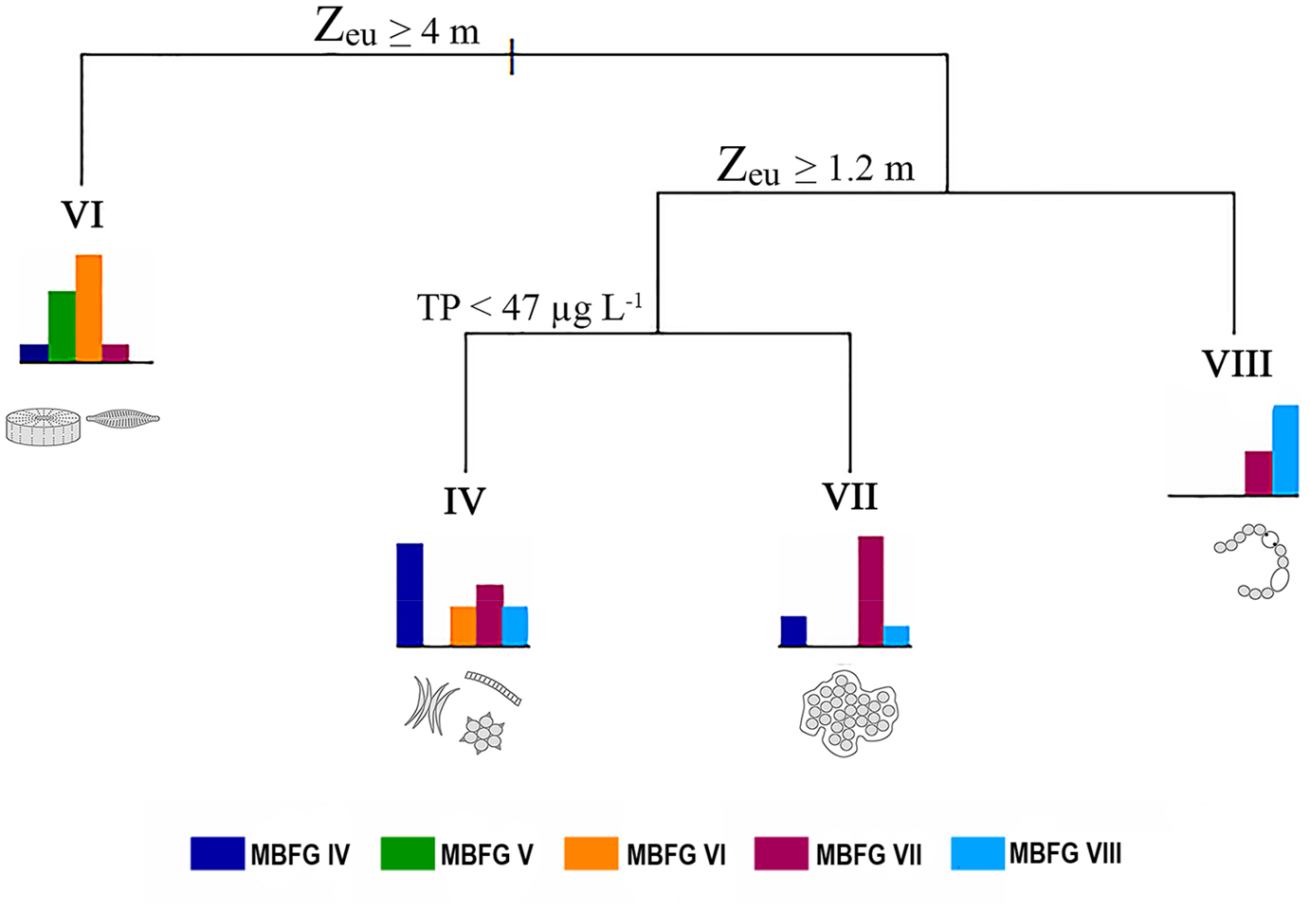
Classification tree displaying the main environmental threshold factor and its value for the dominant MBFG under each specific environmental condition. Only MBFGs with > 70% of the total biomass were included. Among these groups, the MBFG which is most dominated in a certain condition is displayed at the end of each branch with roman numerals. The histogram shows the frequency of MBFG dominance (IV–VIII: left to right) under each of the environmental filters identified by the classification tree. MBFG V was neglected as a class because there was an insufficient amount of data to classify samples

According to the classification tree, *Z*_eu_ and TP were the most important variables to determine environmental thresholds for MBFG dominance (Fig. 6). The classification tree selected *Z*_eu_ for the root node (i.e., the highest node) with a threshold of 4 m, followed by the internal nodes (i.e., a node with successor node or nodes) that subdivided *Z*_eu_ again with a threshold of 1.2 m. Then TP threshold of 47 µg L^−1^ ending in the leaf nodes (i.e., a node without successor). From the left side of the root node, samples with *Z*_eu_ ≥ 4 m were most dominated by MBFG VI, although MBFG V also dominated under this condition (see histograms in Fig. 6). Moving to the right side of the root node, MBFG VIII dominates when *Z*_eu_ < 1.2 m. Meanwhile, MBFG IV and VII dominated when *Z*_eu_ was in the range of 1.2 to 4 m, though MBFG IV dominated when TP was below 47 µg L^−1^ and MBFG VII dominated when TP was above this value. In the classification tree, MBFG V was neglected because there was an insufficient amount of data to classify samples in this class.

### MBFG response to seasonality

**Fig. 7:**
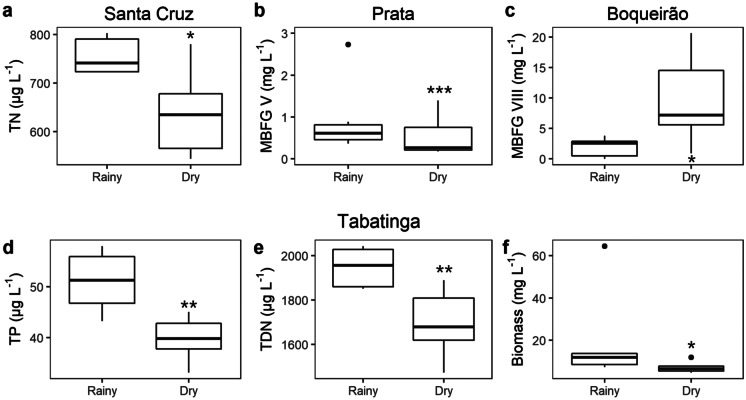
Boxplot of abiotic and biotic variables with significant differences between rainy and dry seasons in lakes; **a** total nitrogen in Santa Cruz, **b** MBFG V biomass in Prata, **c** MBFG VIII biomass in Boqueirão, **d** total phosphorous, **e** total dissolved nitrogen, and **f** phytoplankton biomass in Tabatinga. Bands inside the boxplots represent their medians and vertical bars represent minimum and maximum values. Black dots indicate values that fall outside 1.5 times the interquartile range. All graphs displayed are statistically different with regard to rainy vs. dry seasons (*p*-value < 0.05). Asterisks indicate statistical significance level

The seasonal variability of abiotic and biotic parameters differed among lakes; three of them (Santa Cruz, Prata, and Boqueirão) displayed significant differences between seasons in only one of the variables measured; Santa Cruz Lake had higher values of TN during the rainy season (from January to June) and decreased in the dry season (from July to December; Fig. 7a). Differences in MBFG’s biomass were observed in Prata and Boqueirão Lakes; MBFG V was higher during the rainy season and declined in the dry season in Prata (Fig. 7b), while MBFG VIII was low in the rainy season and increased in the dry season in Boqueirão (Fig. 7c). Meanwhile, in Tabatinga Lake, concentrations of TP (Fig. 7d), TDN (Fig. 7e), and phytoplankton biomass (Fig. 7f) were higher in the rainy season, followed by a decline in the dry season.

**Fig. 8:**
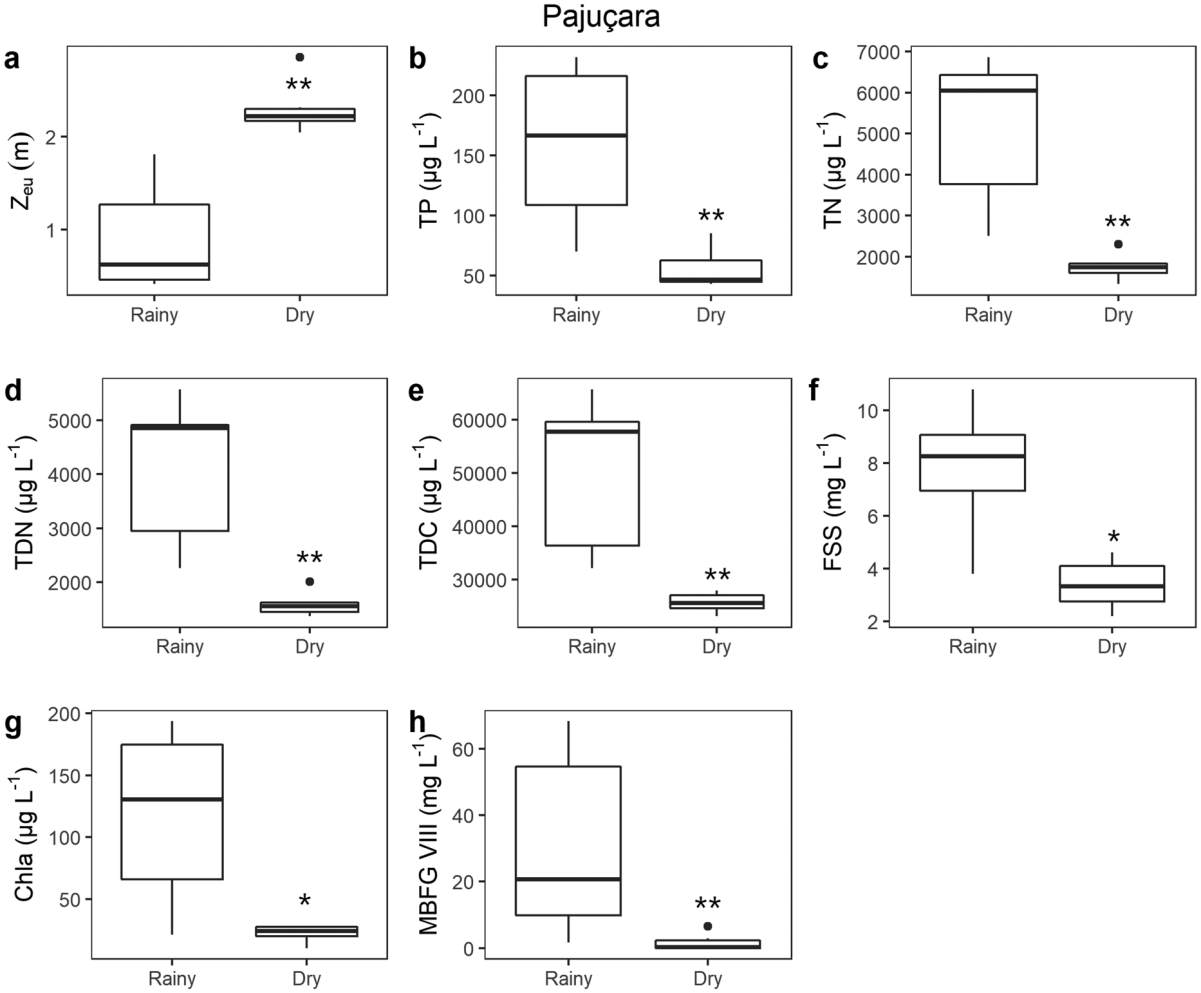
Boxplot of abiotic variables with significant differences between rainy and dry seasons in Pajuçara; **a** euphotic zone depth, **b** total phosphorous, **c** total nitrogen, **d** total dissolved nitrogen, **e** total dissolved carbon, **f** fixed suspended solids, **g** chlorophyll-a, **h** MBFG VIII biomass. Bands inside the boxplots represent their medians and vertical bars represent minimum and maximum values. Black dots indicate values that fall outside 1.5 times the interquartile range. All graphs displayed are statistically different with regard to rainy vs. dry seasons (*p*-value < 0.05). Asterisks indicate statistical significance level

**Fig. 9:**
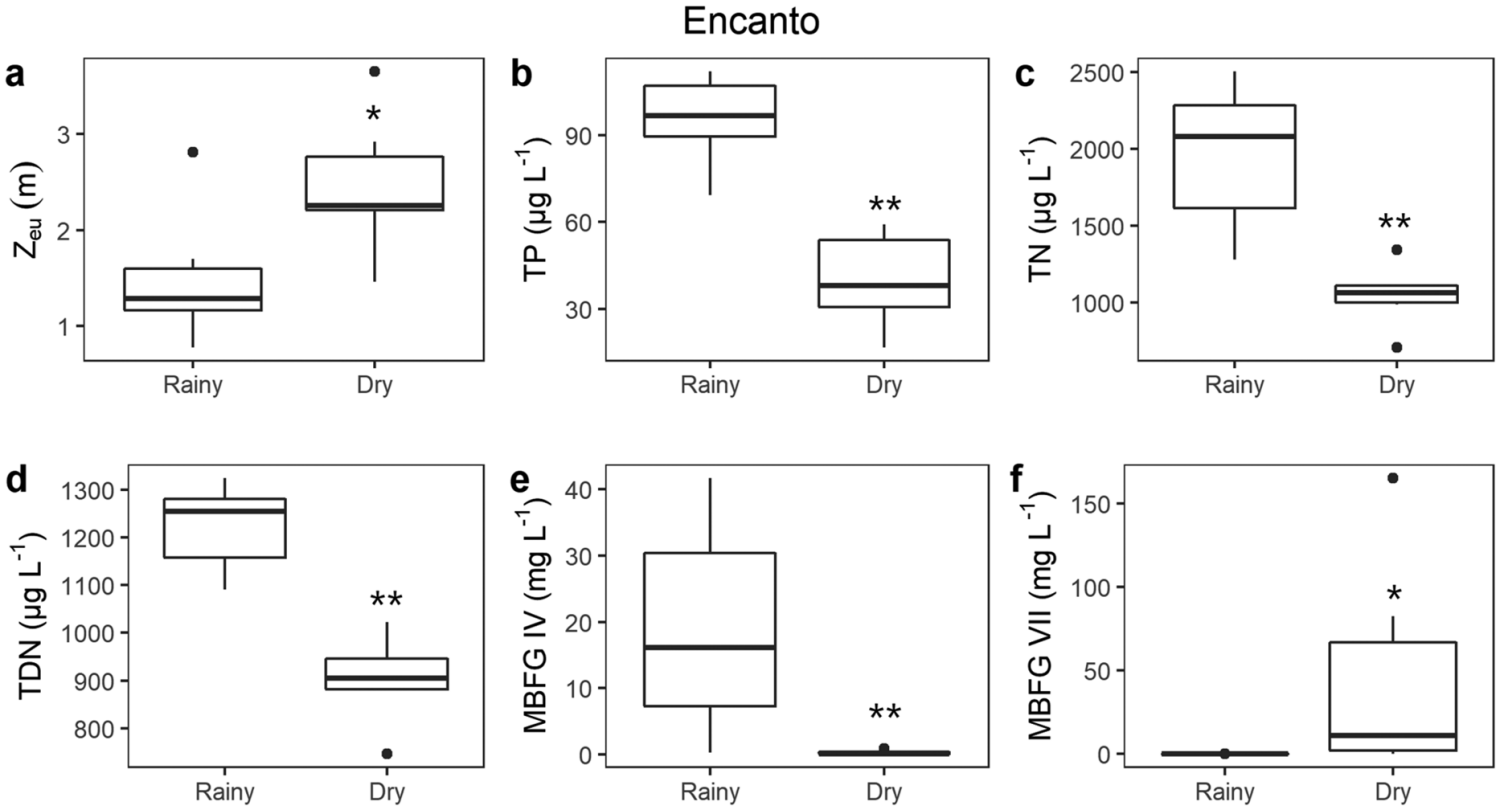
Boxplot of abiotic variables with significant differences between rainy and dry seasons in Encanto;** a** euphotic zone depth, **b** total phosphorous, **c** total nitrogen, **d** total dissolved nitrogen, **e** MBFG IV biomass, and **f** MBFG VII biomass. Bands inside the boxplots represent their medians and vertical bars represent minimum and maximum values. Black dots indicate values that fall outside 1.5 times the interquartile range. All graphs displayed are statistically different with regard to rainy vs. dry seasons (*p*-value < 0.05). Asterisks indicate statistical significance level

The other two lakes presented seasonal environmental differences associated with MBFG selection. In Pajuçara Lake during the rainy season, the *Z*_eu_ (Fig. 8a) was lower, while TP (Fig. 8b), TN (Fig. 8c), TDN (Fig. 8d), TDC (Fig. 8e), FSS (Fig. 8f), Chla (Fig. 8g), and MBFG VIII biomass (Fig. 8h) were higher. Contrarily, in the dry season, the concentration of those variables decreased and the *Z*_eu_ increased. Therefore, Encanto displayed a similar pattern, with a lower *Z*_eu_ (Fig. 9a) and higher concentrations of TP (Fig. 9b), TN (Fig. 9c), TDN (Fig. 9d), MBFG IV biomass (Fig. 9e) during the rainy season, followed by the decrease of nutrient concentration and MBFG IV biomass, along with the increase of *Z*_eu_ and MBFG VII biomass (Fig. 9f) in the dry season. There was no significant difference in water maximum depth between the rainy and dry among lakes (data not shown).

## Discussion

We hypothesized that morphological traits to cope with nutrient limitation are expected to be selected in mesotrophic environments, while traits to overcome light limitation are expected to be selected in more eutrophic lakes. Our findings support this view, as MBFGs IV, V, and VI present features that enable them to thrive in environments with relatively lower nutrient concentrations and were associated with mesotrophic and meso-eutrophic conditions, such as flagella and mixotrophy. Conversely, MBFGs VII and VIII dominated in eutrophic environments, with the high MDL and s/v from MBFG VIII and good buoyant properties on MBFG VII assisting in light interception by the phytoplankton cells.

We also expected that dry and rainy seasons will have a contrasting relationship on environmental filters, such as water depth, the concentration of nutrients and solids, and light availability in selecting distinct MBFGs. Contrary to our expectations, seasonal differences in environmental filters were not a generalized pattern among lakes; for the majority of the lakes (i.e., 4 out 6), seasonality did not influence MBFG selection. Only two of the lakes investigated showed seasonal differences in environmental filters benefiting distinct MBFGs.

The man-made lakes in this study displayed a gradient of phosphorous concentrations and light availability according to their trophic status, which influenced phytoplankton biomass and the selection of morpho-functional traits. Our results highlight the role of light availability and TP concentration as the most important environmental threshold to determine MBFG dominance in tropical semiarid shallow lakes. Our findings corroborate previous results on the influence of trophic status on total phytoplankton biomass, species assemblages, and functional composition (Allende et al., 2019; Izaguirre et al., 2012; Pacheco et al., 2010; Pálffy & Vörös, 2019; Rangel et al., 2016). Furthermore, MBFGs showed a tight association with the trophic states and could be a clear technique to discriminate lakes according to their trophic state.

While nutrient concentrations are assumed to be the major driver of phytoplankton assemblages in oligotrophic and mesotrophic conditions, light availability is of increasing importance towards hypertrophy (Naselli-Flores et al., 2007). Our findings support this view. Species collected in MBFG IV have high growth rates, moderate tolerance to limited resources, and benefit in environments with low light attenuation (Kruk & Segura, 2012; Segura et al., 2013), which make them thrive in less-enriched clear waters or during transitional successional stages (Kruk & Segura, 2012; Magalhães et al., 2020; Reynolds et al., 2002) in agreement with the scenarios found in the mesotrophic and meso-eutrophic lakes, where this group were most frequently found. The environmental thresholds for this group also corroborate this; MBFG IV dominance was related to TP concentrations equal to or below 47 µg L^−1^, which indicates mesotrophic and meso-eutrophic conditions ().

The presence of flagella in MBFG V allows for active resource foraging and these organisms can regulate their position in the water column, along with the production of resting stages that increase their tolerance within unfavorable conditions (Kruk & Segura, 2012; Litchman & Klausmeier, 2008). Moreover, some species in this group are capable of mixotrophy (either phagotrophy or osmotrophy) further increasing their survival chances in environments limited by nutrients and/or light (Costa et al., 2019; Saad et al., 2016). Although nutrient concentrations never fall below values considered limiting for SPR (< 3 µg L^−1^, save 1 month in Santa Cruz) or DIN (< 100 µg L^−1^) (Chorus & Spijkerman, 2021; Reynolds, 2006), mixotrophic species were frequently found in the mesotrophic lakes, Santa Cruz and Prata (*Peridinium* sp., *Cryptomonas* sp., and *Trachelomonas* sp*.*), which may indicate mixotrophy as an important trait in these lakes. Under limiting conditions, mixotrophy represents an important link for the flux of materials via planktonic food webs in freshwater environments (Jones, 2000).

Species belonging to MBFG VI have a siliceous exoskeleton; they lack flagella and have low light requirements, which favor these organisms in turbulent lakes with low light availability (Izaguirre et al., 2012; Kruk et al., 2010; Trindade et al., 2021). In our study, however, this group (mainly small centrales) thrived in a low trophic status environment and a relatively deep *Z*_eu_ (i.e., ≥ 4 m) was the environmental threshold for MBFG VI dominance. Under such conditions in the tropical semiarid region, small centrales species may benefit from diurnal convective mixing within the epilimnion (partial atelomixis) (Barbosa & Padisák, 2003; Souza et al., 2008). Diatoms are known to be good competitors for phosphorus, and this group is frequently associated with low trophic states with low light attenuation in the water column (Bortolini et al., 2019; Kruk & Segura, 2012; Magalhães et al., 2020; Segura et al., 2013).

Conversely, traits to overcome light limitation were selected in eutrophic lakes, and MBFGs VII (large mucilaginous colonies) and VIII (nitrogen-fixing cyanobacteria) dominated in eutrophic environments, yet MBFG VIII dominance was associated with shallower *Z*_eu_ (i.e., ≥ 1.1 m) compared to MBFG VII. Higher trophic states and, subsequently, cyanobacteria overgrowth reduces water transparency, favoring traits typical from MBFG VII, as gas vesicles enable them to migrate in the water column and form dense surface blooms intercepting the influx of light and competitively excluding other phytoplankton species (Huisman et al., 2018). Although MBFG VII dominance was closely related to eutrophic conditions, it also appeared during mesotrophic and meso-eutrophic conditions. Nevertheless, the representative species and biomass differed according to the trophic state. Moreover, the low s/v characteristic from MBFG VII makes them sensitive to low nutrient resources, which may explain the low biomass achieved by this group in mesotrophic conditions compared to other groups. Hence, the inclusion of species from environments with different trophic statuses in MBFG VII limits the effectiveness to assess lake conditions (Bortolini et al., 2016; Pacheco et al., 2010).

MBFG VIII was found exclusively in eutrophic lakes. This group thrives under enriched, turbid water with low transparency (Bonilla et al., 2012; Brasil & Huszar, 2011; Kruk & Segura, 2012; Magalhães et al., 2020; Padisák & Reynolds, 1998). The elongated shape of the algae belonging to this group increases their light-harvesting capacity and competitiveness under low light conditions. Other than the shape, the dominant species from MBFG VIII, *Raphidiopsis raciboskii*, is also a superior shade-tolerant species due to its low light requirement (Burford et al., 2016; Karadžić et al., 2013; Padisák, 1997). In contrast to the dominant species from MBFG VII, *Microcystis* sp. is more light-dependent and grows poorly in turbid environments (Reynolds, 2006; Reynolds et al., 2002), which may explain its absence in shallower *Z*_eu_. Species from MBFG VIII possess heterocytes which can fix atmospheric nitrogen and are widely known to be favored in nitrogen-limited environments (Reynolds et al., 2002; Schindler et al., 2008). However, nitrogen was never limited during the study, and this group was associated with high nitrogen concentrations, implicating that N_2_ fixation is not the trait benefiting these species in eutrophic tropical shallow lakes. Indeed, *Raphidiopsis raciborskii* has already been recognized as an efficient facultative diazotroph, with high competitive strength under fluctuating nitrogen availability (Burford et al., 2016; Hoffman et al., 2011).

Water temperatures were constantly high, with negligible annual temperature oscillations, and did not play a significant role in explaining MBFG variability. Although rainy and dry conditions, instead of temperature, are the natural forcing factors determining seasonality in the tropics (Giani et al., 2020), seasonal (rainy/dry) differences in environmental filters influencing MBFG selection were not a generalized pattern among the studied lakes: of the six lakes investigated, only two showed seasonal differences in environmental filters selecting distinct MBFGs, contrary to what was expected.

The groups MBFGs VIII and MBFG IV benefitted during the rainy season, at Pajucara and Encanto Lakes, respectively, when the higher concentration of nutrients (phosphorous and nitrogen), FSS and Chla, resulted in a reduced *Z*_eu_. In Encanto Lake, the species succeeding the most was *Pseudanabaena* sp*.* (MBFG VI), which thrives in a low light environment, favored by its elongated shape, similarly to MBFGs III and VIII. This pattern was followed by the collapse of MBFG VIII in the dry season, when the concentration of nutrients decreased, leading to an increase of the *Z*_eu_, evidencing the association of MBFG VIII with enriched, turbid, and low water transparency conditions (Bonilla et al., 2012; Brasil & Huszar, 2011; Kruk & Segura, 2012; Magalhães et al., 2020; Padisák & Reynolds, 1998). At Encanto Lake, MBFG VII benefited from the decline of nutrients and increase of euphotic zone depth which is in line because this group was already linked with low light attenuation conditions (Kruk & Segura, 2012). Additionally, the higher concentration of TP and TDN during the rainy season in Tabatinga led to high phytoplankton biomass; however, it was not related to the selection of any particular MBFG.

The other three lakes did not show a clear seasonal pattern regarding environmental filters and MBFG selection; for them, seasonal differences were observed only on environmental filters or MBFGs. In Santa Cruz, the higher total nitrogen concentration in the rainy season did not reflect the seasonal difference in MBFGs composition. Despite MBFG V and VIII differed among seasons in Prata and Boqueirão, respectively, these changes were not associated with the seasonality of environmental filters measured in this study.

Our results showed that not all lakes presented seasonal environmental differences associated with MBFG selection, which indicate that the response to seasonality may be lake-dependent. Nevertheless, when seasonal differences were observed on environmental filters, the rainy season was always linked with higher concentrations of nutrients and suspended solids, as well as a lower *Z*_eu_ when compared to the dry season. Yet, this does not refute previous studies in tropical semiarid regions showing that the rainy season dilutes nutrient concentration increasing *Z*_eu_, while the dry season triggers eutrophication and cyanobacteria dominance (Braga & Becker, 2020; Brasil et al., 2016; Costa et al., 2016; Medeiros et al., 2015). Instead, it highlights that effects other than nutrient dilution may be expected when the rainy season is not associated with the increase in water depth or volume. Indeed, less intense events of rain may increase nutrient concentration via short-term runoff events that do not increase water depth or dilution, in contrast to long rainy periods (Stockwell et al., 2020). Although rainfall in 2017 was in general above the average compared to the prolonged drought (2012–2016), it was still below the historical average and did not result in a significant difference in water level between the rainy and dry seasons. Furthermore, the majority of lakes (i.e., 5 out of 6) were very shallow and rain events may resuspend sediment via increasing water column mixing (Marion et al., 2017; Søndergaard et al., 2003).

The eutrophic lakes (Encanto, Pajuçara, and Boqueirão) were previously characterized by a perennial dominance of cyanobacteria (Vanderley et al., 2021). Hence, the dry season itself was in general not related to cyanobacteria dominance as reported by previous studies (Braga & Becker, 2020; Brasil et al., 2016; Costa et al., 2016; Medeiros et al., 2015). Yet, the dry and rainy seasons benefited distinct morpho-functional traits of cyanobacteria in these lakes. The identification of environmental filters and thresholds benefiting different cyanobacteria’s morpho-functional traits is essential to understanding their dynamic under perennial bloom to mitigate. Moreover, droughts are becoming more intense in the Brazilian semiarid region and a further increase in rainfall deficit and aridity are expected for the second half of the twenty-first century (Marengo et al., 2017, 2018, 2020). As consequence, profound effects on water quantity and quality have already been reported via the vulnerability of shallow tropical lakes to the role of drought in triggering eutrophication and cyanobacterial blooms (Braga & Becker, 2020; Braga et al., 2015; Brasil et al., 2016; Costa et al., 2016; Figueiredo & Becker, 2018; Medeiros et al., 2015). A review on cyanobacterial blooms in this region also reported an increase in intensity and duration of such blooms, along with the occurrence of toxic events (Amorim et al., 2020; Moura et al., 2018). An increase of saxitoxin in the drinking water supply during this prolonged drought was also linked to the highest incidence of microcephaly associated with the Zika virus in Brazil (Pedrosa et al., 2020), highlighting a great threat to human health.

The application of functional approaches and the identification of its environmental thresholds improves our knowledge of environmental drivers structuring the phytoplankton assemblage and increases the capacity to manage and mitigate water quality under the ongoing environmental changes. Our results showed MBFG is an effective tool to assess lake conditions and the dynamic of cyanobacterial morpho-functional traits under perennial bloom conditions, which is in line with previous studies recommending this approach for biomonitoring proposes (Cupertino et al., 2019; Izaguirre et al., 2012; Lyche-Solheim et al., 2013; Rangel et al., 2016). Although toxin analyses are irreplaceable, MBFG could be used to predict potential toxic events via the monitoring of MBFGs containing toxin producer species reducing the number of samples to analyze, and the labor costs of biomonitoring (Rangel et al., 2016). However, this proposal needs to be validated, as being an easy system to apply, ideal for handling a large number of samples, without demanding further knowledge of autecology and particular taxonomic skills, especially for species whose traits properties have been poorly known yet (Hu et al., 2013; Izaguirre et al., 2012; Kruk et al., 2010).

Like all functional approaches, the MBFG (Kruk et al., 2010) has advantages and limitations. It is beneficial for being objective and easy to apply; however, this simplification reduces its sensitivity to capture important phytoplankton traits that would be necessary to understand the functionality of the system, such as mixotrophy, potential nitrogen fixation, and inclusion of species indicating other trait selective drivers. Overall, morphological traits associated with trophic status and the identification of environmental thresholds for their dominance via machine learning (CART) set a precedent for its use and could offer a handy tool, especially for environmental agencies, to manage and even predict cyanobacterial blooms when applied with shared or integrate data of cyanobacteria in the semiarid regions.

## Conclusion

Morpho-functional traits showed a tight association with the gradient of light and phosphorous concentrations found in the shallow, man-made lakes in a semiarid region. Hence, TP and *Z*_eu_ were the main drivers determining the environmental threshold for MBFG dominance. The mesotrophic and meso-eutrophic lakes selected phytoplankton species with traits to overcome low nutrient conditions (MBFGs VI, V, and VI). Meanwhile, in eutrophic environments, traits to overcome low light conditions were selected (MBFG VII and VIII). Furthermore, the MBFG approach showed to be a clear technique to discriminate lakes according to their trophic state, which along with the environmental thresholds for MBFG dominance offers a handy tool for biomonitoring cyanobacterial blooms in semiarid regions, especially for environmental agencies. Contrary to our expectations, the seasonal pattern of environmental filters influencing the selection of morpho-functional traits was not general, yet when seasonal environmental differences were observed, the rainy season was associated with a higher concentration of nutrients, suspended solids, and reduced *Z*_eu_ compared to the dry season. Although the effects of seasonality may affect environmental filters and MBFG selection in tropical lowland lakes, our results show that the effects of seasonality varied across lakes and highlight eutrophication as the main environmental factor for MBFG selection, which taken together suggests reduced seasonality effects during drought periods in the tropics.

## Data Availability

The datasets generated during and/or analyzed during the current study are available from the corresponding author on reasonable request.
